# CD14 dictates differential activation of mesenchymal stromal cells through AKT, NF-κB and P38 signals

**DOI:** 10.1042/BSR20190807

**Published:** 2019-07-05

**Authors:** Menghui Jiang, Tianlin Gao, Yuansheng Liu, Xue Cao, Yanting Li, Jianyu Li, Yuanjiao Liu, Jinmei Piao

**Affiliations:** School of Public Health, Qingdao University, Qingdao, China

**Keywords:** CD14, Mesenchymal stroma cells, TLRs

## Abstract

Mesenchymal stromal cells (MSCs) widely exist in many tissues and have multiple differentiation potential and immunomodulatory capacities. Recently, MSCs have become promising tools for the treatment of various degenerative disorders and autoimmune diseases. The properties of MSCs could be modified in different microenvironments. Thus, it is important to explore the factors controlling MSC function. The presence of Toll-like receptors (TLRs) in MSCs was demonstrated according to previous studies. Consistently, we also illustrated the expression of TLRs in both murine and human MSCs, and displayed that the expression patterns of TLRs in MSCs from different sources. Furthermore, we explored the role of TLR and TLR signaling pathway in MSCs. Interestingly, activation of TLR4-induced expression of cytokines and some specific genes in MSCs. However, MSCs retained much lower mRNA level compared with macrophages. We explored the expression of CD14 in MSCs from different sources, which played a vital role in TLR4 signaling pathway, and found that MSCs are almost negative for CD14. Moreover, only partial activation of TLR4 signaling pathway was observed in MSCs, with no activation of AKT, NF-κB and P38. Here, in the study we defined TLR expression, function and activation in MSCs, which is critical for designing MSC-based therapies.

## Introduction

Mesenchymal stromal cells (MSCs) reside in multiple tissues of mesodermal origin, including bone marrow [1], adipose tissue [2], umbilical cord [3], dental pulp [4] and so on. MSCs are defined by the ability to self-renew and to differentiate into various cell lineages, such as osteoblasts, adipocytes and chondrocytes, which endow MSCs the ability to repair tissues [5]. Actually, MSCs have already been permitted to be used in clinical to treat tissue injuries and degenerating diseases. In addition, recently MSCs are found to preserve immunosuppressive capability [6], which allow MSCs to become a promising tool for the treatment of immune disorders. However, the detailed mechanisms involved in MSC immunoregulatory capacity are still elusive and need more investigation. Various soluble factors have been identified to regulate MSC immunoregulatory capacity, such as indoleamine 2,3-dioxygenase (IDO), nitric oxide (NO), prostanglandin-E2 (PGE2), interleukin (IL)-10, transforming growth factor (TGF)-β1, hepatocyte growth factor (HGF), heme oxygenase-1 (HO-1) and HLA-G5 [7]. Among these factors, for human MSCs IDO plays a pivotal role in mediating immunosuppression, whereas NO is the main mediator for mouse MSCs [8].

Interestingly, human and mouse MSCs could not exert immunomodulating capacity spontaneously, but function when treated with inflammatory cytokines, such as IFNγ, TNFα, IL1α and IL1β [9]. These cytokine combinations induce high expression of IDO and iNOS in human MSCs and mouse MSCs separately [8]. In addition, the immunosuppression of MSCs could also be influenced by Toll-like receptors (TLRs). TLRs are a family of type I transmembrane receptor, which contain 11 members in human and 13 members in mouse separately [10]. Moreover, the recognition of specific ligands by TLRs will trigger inflammation in both infectious and non-infectious diseases. TLRs are widely expressed by the immune cells; therefore, the studies about TLRs mainly focus on immune cells initially. However, TLRs are found expressed in MSCs, among which TLR3 and TLR4 are found highly expressed in human bone marrow-derived MSCs (BMSCs) [11]. TLR ligands are also found to be involved in modulating MSC immunosuppression, however, it is still inconclusive that TLR ligands reduce or enhance MSC immunosuppressive capacity.

TLR activation induces intracellular signals which lead to the expression of specific genes. When combined with ligands, TLRs formed dimers and often utilize CD14 to assist in pathogen recognition. Specifically, CD14 combines with TLR4 dimer to transduce activation signals, and increase the potency of the response to LPS, as a high-affinity receptor for LPS on cells to facilitate LPS signaling [12]. CD14 acts as a co-receptor, exhibiting many characteristics of a pattern-recognition receptor, which is expressed mainly by macrophages, neutrophils and dendritic cells. Whereas, MSCs are considered as deficiency of CD14 expression, which is a criteria for defining MSCs [13]. Interestingly, recently <5% proportion of MSCs are found to express CD14 after analysis by flow cytometry [14]. Here, we find that that both human and mouse MSCs expressed various TLRs and slight level of CD14, compared with monocytes/macrophages. Therefore, identifying the expression of CD14 in MSCs and comparing the difference of TLR signals in MSCs and immune cells become an important question.

In the current study, we identify whether decreased TLR4 signals is due to the less expression of CD14. We show here that lack of CD14 abrogate the phosphorylation of P38, P65 and AKT molecules. Thus, we found compelling evidence that CD14 expression in MSCs plays an important role in response to LPS and in regulating TLR4 signaling pathway.

## Materials and methods

### Isolation and culture of MSCs

Primary human MSCs derived from bone marrow, adipose tissue and umbilical cord were isolated from normal adult volunteers according to previous protocol [8]. Murine bone marrow MSCs were collected as described previously according to our lab protocol. Briefly, mice bone marrow was flushed from femur, the bone marrow suspension was concentrated, plated in flask and cultured in the incubator. Both human and murine MSCs were cultured in DMEM containing 10% FBS and penicillin/streptomycin (all from Gibco, Carlsbad, CA, U.S.A.).

### Reagents

Reagents for inducing MSC differentiation, including 3-isobutyl-1-methylxanthine, l-ascorbic acid, indomethacin, dexamethasone, insulin, β-glycerophosphate, Alizarin Red S, and Oil Red O were purchased from Sigma–Aldrich (St. Louis, MO, U.S.A.). Antibodies used in Western blotting analysis were: AKT, pAKT, pP38, pP65, pJNK, pERK and GAPDH (Cell Signaling Technology, Danvers, MA, U.S.A.). Fluorescence antibodies used in flow cytometry were: CD45, CD31, F4/80, MHCII I-A, MHCI KbDb, Sca1 and CD44 (eBioscience, Thermo Fisher, U.S.A.).

### Flow cytometry

To characterize the MSCs identity, the cells were washed twice by PBS, and then pre-blocked by 2% FBS in PBS. After mixing with specific antibodies, the cell suspension was incubated for 30 min on ice. The cell pellets were washed for three times by PBS, and then were analyzed by FACS Calibur flow cytometer (BD, San Jose, CA, U.S.A.). The results were analyzed by FlowJo software.

### Differentiation assays

For osteogenic differentiation, MSCs were cultured in high glucose medium supplemented with 10 nM dexamethasone, 0.2 mM ascorbic acid and 10 mM b-glycerophosphate. To induce adipogenesis, MSCs were cultured in medium containing 100 nM dexamethasone, 0.5 mM 3-isobutyl-1-methylxanthine, 10 mM insulin and 200 mM indomethacin. After osteogenesis, mineral formation was stained for 15 min with 2% Alizarin Red S solution at room temperature. Lipid droplets in MSCs were stained for 30 min with Oil Red O at room temperature. Finally, the cells were washed three times with diluted water and photographed.

### Real-time PCR

Total RNA was extracted with TRIzol, and was reversed into cDNA according to manual. The profile of gene expression was performed using SYBR Green, and analyzed with the ABI7500 detector instrument. Gene expression levels were calculated using β-actin as control. The applied primers were listed in Supplementary Table S1.

### Western blotting

MSCs were lysed on ice for 30 min with RIPA buffer (Beyotime, China) supplemented with protease inhibitor cocktail (Roche Applied Science, Mannheim, Germany). After boiling, the protein extract (40 μg) was loaded on to 10% SDS/PAGE gels and transferred on to nitrocellulose membrane (Millipore). The protein amount was detected with ECL assay according to manufacturer’s manual. In brief, after coated with primary and secondary antibodies, the nitrocellulose membrane was incubated with HRP ubstrates and the fluoresence signal was detected.

### Statistical analysis

Prism 7.0 software (GraphPad Software, Inc.) was applied to analyze the data, which was used for graphs drawing and statistical significance analysis. The statistical significance is reported as follows: ns, not significant; *P<0.05; **P<0.01; ***P<0.001.

## Results

### Characterization of murine bone marrow MSCs

**Figure 1:**
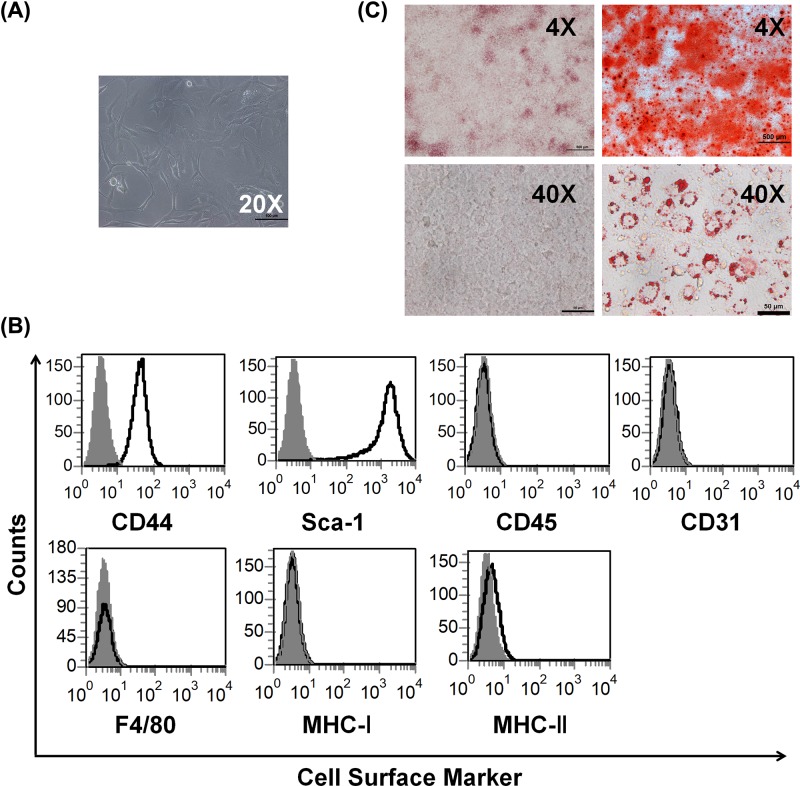
Characterization of murine MSCs (**A**) Morphology of murine MSCs (20× magnification). (**B**) MSCs were stained with specific surface markers or control antibodies and subjected to flow cytometry analysis. Cell surface markers, red; isotype controls, black. (**C**) MSCs were cultured with induction medium for indicated time to differentiation. Differentiation into osteoblasts and adipocytes were determined by Alizarin Red staining and Oil Red O staining separately (4×, 40× magnification, respectively).

Previously various different protocols were reported in terms of isolation, characterization and expansion of MSCs [15]. BMSCs were considered the best cell source, which were the most widely studied, and usually taken as a standard for the comparison of MSCs from other sources. Therefore, murine MSCs were isolated from the femur of 6–8 weeks old mice according to the protocol established by our lab. Later, bone marrow-derived MSCs were cultured in complete DMEM for selection of MSCs. After culturing for several continuous passages, MSCs displayed a stable fibroblast-like phenotype and were used for further experimentation (Figure 1A). To further characterize MSCs, the cells were analyzed by flow cytometry for expression of specific phenotypic markers of MSCs [16], and as evidenced the cells were uniformly and strong positive for MSC markers, Sca-1 and CD44, were negative for CD45, CD31, F4/80, MHC-I and MHC-II (Figure 1B). Moreover, to better characterize MSCs, we further detected the ability of MSC differentiation, and found that MSCs exhibited *in vitro* competence to differentiate into osteoblasts and adipocytes confirmed by Alizarin Red staining and Oil Red O staining, respectively (Figure 1C). These results indicate that we isolated the exactly right MSCs.

### Murine MSCs expressed various TLRs, the most high expression being TLR4

**Figure 2:**
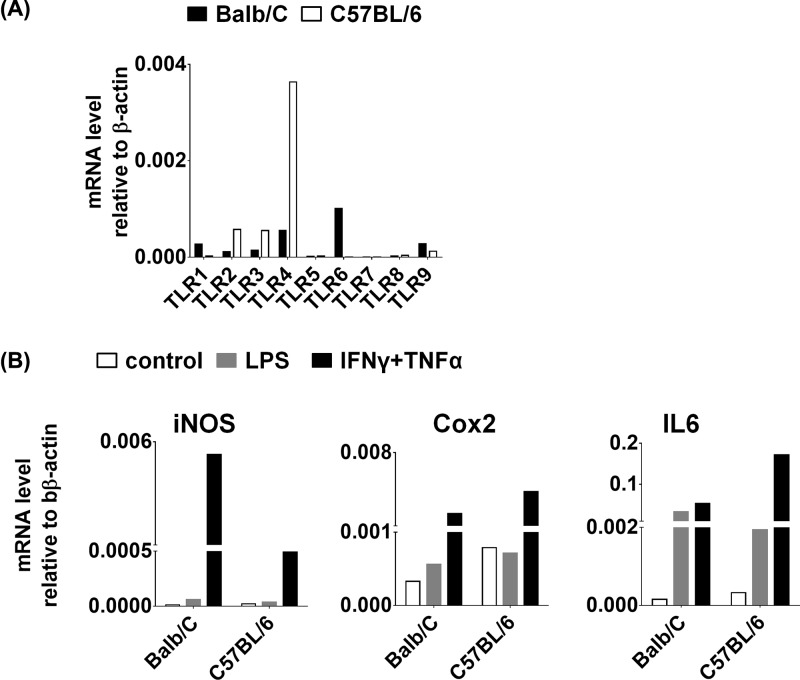
Characterization of TLR expression in murine MSCs (**A**) The mRNA levels of TLR-1–TLR-9 were assessed by real-time PCR in two different murine MSCs, which were isolated from Balb/C and C57BL/6, respectively. (**B**) Both Balb/C and C57BL/6 MSCs were treated with LPS (gray), and IFNγ plus TNFα combination (black) for 1 day. After that, the mRNA levels of iNOS, Cox2 and IL6 were determined by real-time PCR in Balb/C and C57BL/6 MSCs, respectively.

Previously, TLRs were found to be widely expressed on various immune cells, such as macrophages and dendritic cells and so on [17]. Therefore, studies about TLRs were mainly focused on these immune cells before. Interestingly, TLRs expression were also found on MSCs recently [18]. Therefore, we next examined the expression of various TLRs in MSCs derived from Balb/C and C57BL/6 mice by QPCR, and found that murine MSCs expressed TLR1–TLR9, among which the expression of TLR4 was the highest (Figure 2A). MSCs acquired immunomodulation properties after being primed with IFNγ and IL1, TNFα or LPS [18]. Therefore, we treated both types of murine MSCs with LPS, taking IFNγ plus TNFα treatment as positive control, and found that IFNγ plus TNFα stimulation induced high expression iNOS, Cox2 and IL6 (Figure 2B). Whereas, LPS stimulus only induced a little bit of expression of IL6 in Balb/C MSCs, with iNOS and Cox2 expression no induction (Figure 2B).

**Figure 3:**
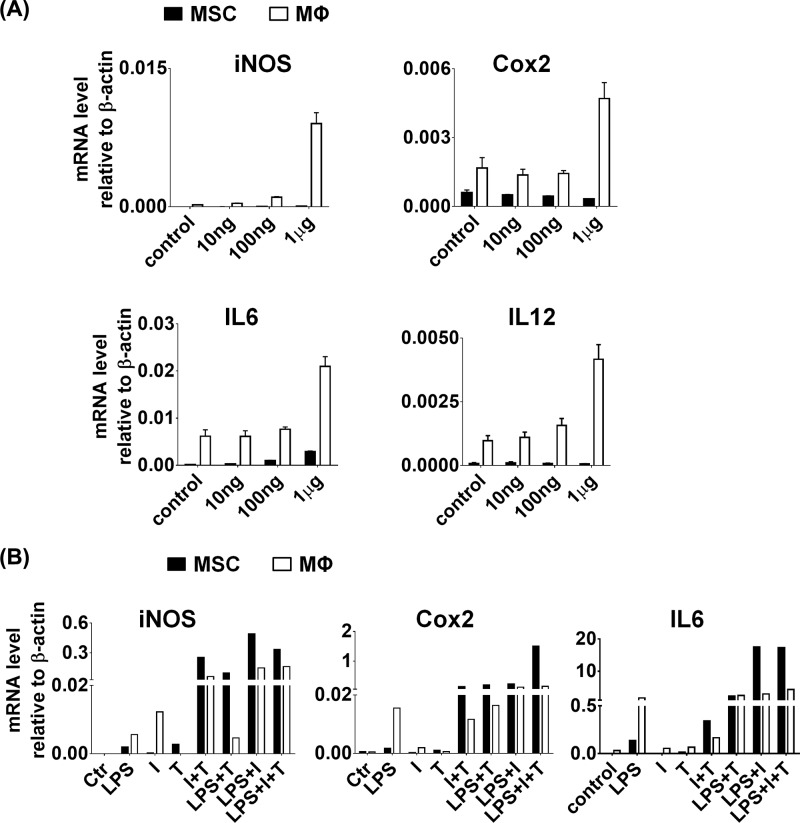
Murine MSCs were less sensitive in response to LPS compared with macrophages (**A**) Murine MSCs and macrophages were stimulated with different concentrations (10 ng/ml, 100 ng/ml, 1 μg/ml) of LPS for 1 day. And the mRNA levels of iNOS, Cox2, IL6 and IL12 were detected by real-time PCR. (**B**) Murine MSCs and macrophages were treated with different cytokines alone or cytokine combinations for 1 day. And the mRNA levels of iNOS, Cox2 and IL6 were detected by real-time PCR.

Considering macrophages retain high expression of TLR4, we stimulated murine MSCs and macrophages with different concentrations of LPS. The results showed that high-dose (1 μg/ml) LPS treatment induced obvious increase in iNOS, Cox2, IL6 and IL12 expression in macrophages, whereas under LPS treatment the expression of these genes in MSCs had no big difference compared with macrophages (Figure 3A). Furthermore, we treated MSCs and macrophages with LPS, IFNγ, TNFα and various cytokine combinations, and observed that LPS alone, and LPS plus IFNγ, TNFα or together all induced high expression of iNOS, Cox2 and IL6 in macrophages, whereas cytokine combinations all induced high expression of iNOS, Cox2 and IL6 in MSCs but not LPS alone (Figure 3B). These results indicated that MSCs were less sensitive in response to LPS compared with macrophages.

### TLR stimulation in human-derived MSCs triggers the induction of cytokines and chemokines, but not IDO, Cox2 and TSG6

**Figure 4:**
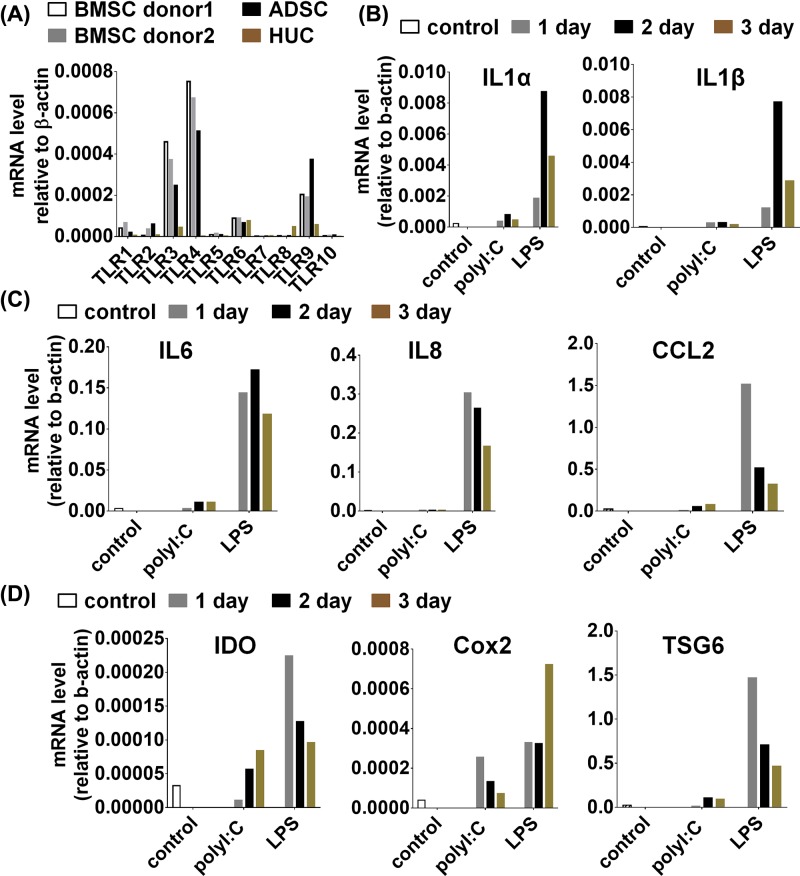
Human MSCs also expressed TLRs and LPS induced expression of cytokines and specific genes (**A**) The mRNA levels of TLR-1 to TLR-10 were assessed by real-time PCR in different human MSCs, origin from bone marrow, adipose tissue and umbilical cord. Human BMSCs were treated with polyI:C and LPS for indicated times. After that, the mRNA levels of IL1α, IL1β (**B**) IL6, IL8, CCL2 (**C**) IDO, Cox2 and TSG6 (**D**) were analyzed by real-time PCR.

We also analyzed the expression of various TLRs in human MSCs derived from bone marrow, adipose tissue and umbilical cord, and found that all types of MSCs, except umbilical cord-derived MSCs (HUC), expressed similar levels of TLR1–TLR10, among which the highest expression level was TLR3 and TLR4 (Figure 4A). Bone marrow-derived MSCs (BMSCs) expressed the highest level of TLR3 and TLR4 among these types of MSCs (Figure 4A). Therefore, to investigate the effect of TLR stimulation in hMSCs, BMSCs were stimulated with polyI:C and LPS for up to 3 days, and the expression profiles of various genes were determined by QPCR. At the mRNA level, only LPS stimulus induced markedly higher level of inflammatory cytokines, including IL1α, IL1β, IL6, IL8 and CCL2 in comparison with polyI:C stimulus (Figure 4B,C). In addition, LPS stimulus induced much higher level of IDO, Cox2 and TSG6 than polyI:C stimulus did (Figure 4D). The results indicated that MSCs appears to prefer express proinflammatory factors such as IL1α, IL1β, IL6, IL8 and CCL2 under LPS stimulus but not polyI:C stimulus.

**Figure 5:**
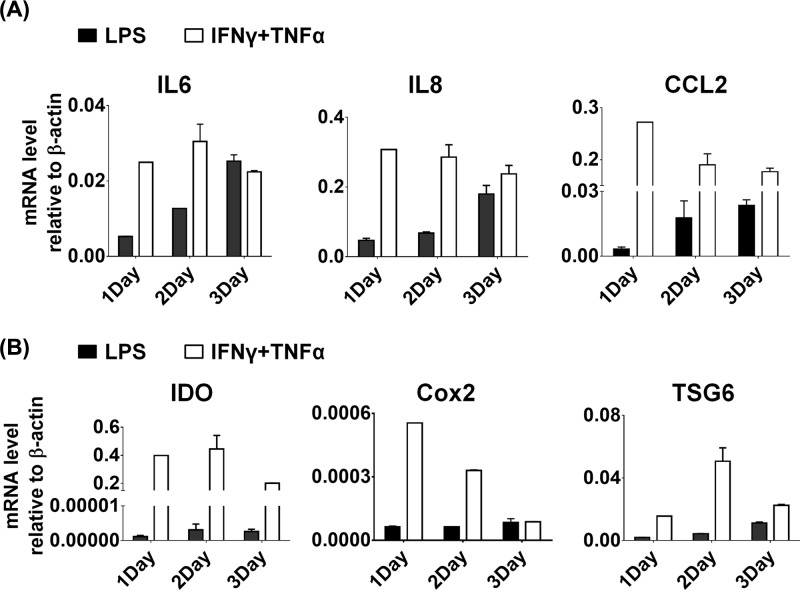
Human MSCs were treated with LPS and cytokine combination Human BMSCs were treated with LPS and IFNγ plus TNFα combination for indicated times. After that, the mRNA levels of IL6, IL8, CCL2 (**A**) IDO, Cox2 and TSG6 (**B**) were analyzed by real-time PCR.

To check the activated state of BMSCs treated with LPS, BMSCs were stimulated with LPS and IFNγ plus TNFα individually at the same time. After that, the gene expression was analyzed by QPCR. We found that LPS stimulus induced comparative amounts of inflammatory cytokines, including IL6, IL8 and CCL2, in comparison with IFNγ plus TNFα treatment (Figure 5A). However, BMSCs exhibited tremendous lower levels of IDO, Cox2 and TSG6 under LPS stimulus, compared with IFNγ plus TNFα treatment (Figure 5B). Overall, these results indicate that LPS could activate the downstream gene expression in MSCs, but could not fully activate gene expression like IFNγ plus TNFα treatment.

### CD14 deficiency led to partial TLRs stimulation inducing specific activation of downstream signals in MSCs

**Figure 6:**
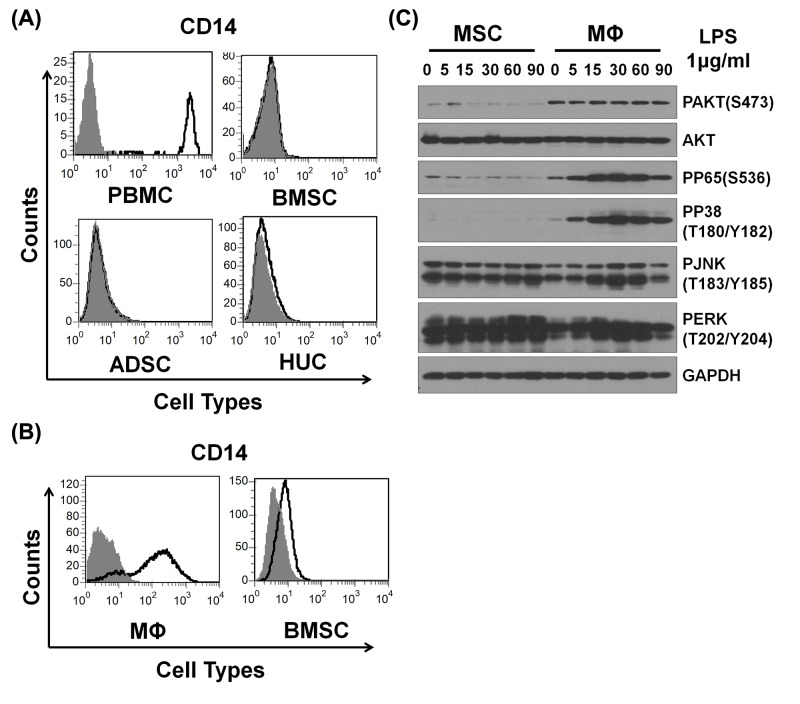
CD14 deficiency led to partial TLRs stimulation in MSCs (**A**) Human PBMC and different sources of MSCs were stained with CD14 and isotype control and analyzed by flow cytometry. CD14, black; isotype control, gray. (**B**) Murine peritoneal macrophages and BMSCs were also treated like (A). (**C**) Murine peritoneal macrophages and BMSCs were stimulated with LPS (1 μg/ml) for different times. And the molecular signals were analyzed by Western blot.

CD14 was well known to increase the sensitivity of TLR4 to LPS by 100–10000 folds [19]. To examine whether CD14 was involved in the process, the expression of CD14 was analyzed in MSCs and monocytes derived from human and mice by FACS. As shown in the results, human-derived PBMC exhibited high expression of CD14, whereas human MSCs derived from bone marrow, adipose tissue and umbilical cord all were almost negative for CD14 expression (Figure 6A). Similarly, murine macrophage also displayed high expression of CD14, whereas murine MSCs were also almost negative for CD14 expression (Figure 6B). Therefore, we inferred that the lack of CD14 in MSCs led to partly activation of TLR4 downstream signaling pathway. To investigate mechanisms of TLR4 partly activation, the protein level of key molecules were examined by Western blot in MSCs and macrophages after treated with LPS for different time points. We observed that pAKT (Ser^473^), pP65 and pP38 were activated in macrophages but not in MSCs, whereas pJNK and pERK displayed no obvious difference (Figure 6C). Thus, our data together with previous studies demonstrate that CD14 deficiency in human and murine MSCs inhibits TLR4 sensitivity to LPS via pAKT (Ser^473^), pP65 and pP38 signals.

## Discussion

MSCs existed in almost all tissues, and recently MSCs have already become an important therapeutic strategy for various proinflammatory and autoimmune diseases, as a result of their powerful immunomodulatory properties and differentiation ability [20,21]. More understanding of the factors and mechanisms regulating their immunomodulatory ability were crucial, which could allow us to operate MSCs for therapy in future clinical treatment. In the study, we reported that TLR4 activation in MSCs led to increased expression of cytokines and chemokines, and partial activation of established TLR signaling pathways.

Previously, studies about TLRs mainly focused on their function and signaling mechanism in various immune cells [22,23]. However, recent reports have illustrated that MSCs express a series of TLRs [18,24,25]. Therefore, the expression of TLRs in murine MSCs and human-derived MSCs from different origins was determined. Consistent with previous reports, we also found that on the mRNA level MSCs expressed various TLRs, with the abundant expression of TLR3 and TLR4. Macrophage is considered as the classical cell for TLR studies. Therefore, in addition, we compared the gene expression of murine MSCs and macrophages under LPS and different cytokine combinations treatment. When the gene expression profiles were analyzed by QPCR after treatment, iNOS, Cox2, IL6 and IL12 were found to be notably increased in macrophages at the mRNA level, while only IL6 was found to be slightly increased in MSCs. At the same time, we stimulated human-derived BMSCs with poly(I:C) and LPS for indicated time. We found that cytokines, chemokines, IDO, Cox2 and TSG6 strikingly increased at the mRNA level in human BMSCs under LPS treatment, but not poly(I:C) treatment. Therefore, we showed that LPS treatment induced IL6 expression in murine MSCs and IDO, TSG6 and IL6 expression in hMSCs, respectively.

The expression of specific genes is regulated by a series of signals under TLR ligands stimulation. Most reports have shown that the presence of CD14 was necessary for TLR4 in response to LPS [26,27], and that CD14 presence increased the potency of response to LPS [12,28]. The crystal structure of CD14 provided evidence that CD14 itself kept closely with the distance between LPS molecules and catalyzed receptor dimerization [29,30]. Therefore, the presence of CD14 was inferred as an essential factor allowing TLR4 signals to switch into a ‘full activation’ mode. Recently, some reports demonstrated that poly(I:C) and LPS induces activation of NF-kB, MAPKs and AKT signaling pathways in MSCs [31]. The activation state of these pathways was associated with the induction cytokines and specific genes in MSCs. Therefore, to verify the role of CD14 in MSCs, utilizing FACS the expression of CD14 in human BMSCs, ADSCs and HUCs was compared with human PBMCs, while murine BMSCs was compared with murine peritoneal macrophages. The results displayed that all human MSCs derived from various sources and murine MSCs were negative for CD14. To further understand the TLR4 downstream molecular signaling in MSCs, we investigated TLRs and their downstream signals. Various studies had shown that NF-κB signal was important for activation of TLR signaling pathway [32–37]. When treated with LPS, murine MSCs displayed remarkably less phosphorylation of AKT, NF-κB and P38 compared with macrophages, whereas the phosphorylation of JNK and ERK was comparable between MSCs and macrophages. Therefore, we proposed that lack of CD14 in MSCs led to ‘partial activation’ mode of TLR4 signaling pathway, thereby inducing some specific genes expression.

In conclusion, our findings revealed that LPS treatment induced slightly expression of cytokines and specific genes in murine and human MSCs. The expression was lower compared with macrophages due to absence of CD14 in MSCs, which led to ‘partial activation’ mode of TLR4 signaling pathway. Our findings provided new insights into better defining strategies of cell-based therapies for autoimmune disorders.

## Concluding remarks

We report that both human and murine MSCs express various TLRs on the membranes, among which the level of TLR4 is the highest. Interestingly, upon LPS stimulus MSCs express lower level of specific genes compared with macrophages. Moreover, we found that both human and murine MSCs almost negative for CD14 expression. Importantly, only partial activation of TLR4 signaling pathway was observed in MSCs, with no activation of AKT, NF-κB and P38.

In summary, our discoveries not only reveal a physiological role of CD14 in modulating activation of MSCs but also provide new insights into the importance of comprehensive immunomodulatory capacity of MSCs. This work has important implications for designing new therapeutic strategies for MSCs clinical application.

## Supporting information

**Supplementary Table S1:** 

## Abbreviations

ADSC: adipose tissue derived MSC
Cox2: Cyclooxygenase 2
HUC: human umbilical cord derived MSC
IDO: indoleamine 2,3-dioxygenase
IL: interleukin
MSC: mesenchymal stromal cell
NO: nitric oxide
TLR: Toll-like receptor
TSG6: TNF-stimulated gene 6 protein
