# Supplementary material for: CD14 dictates differential activation of mesenchymal stromal cells through AKT, NF-κB and P38 signals
Source: Biosci Rep. 2019 Jul 5;39(7):BSR20190807. doi: 10.1042/BSR20190807 (PMC6609595; doi:10.1042/BSR20190807)
Supplement: Supplementary file 1 [file bsr20190807_supp1.pdf]

Table S1

Human TLR1	F: CCACGTTCCTAAAGACCTATCCC<br>R: CCAAGTGCTTGAGGTTCACAG
Human TLR2	F: ATCCTCCAATCAGGCTTCTCT<br>R: GGACAGGTCAAGGCTTTTTACA
Human TLR3	F: TTGCCTTGTATCTACTTTTGGGG<br>R: TCAACACTGTTATGTTTGTGGGT
Human TLR4	F: TTTGGACAGTTTCCCACATTGA<br>R: AAGCATTCCCACCTTTGTTGG
Human TLR5	F: GCCGGTCCTGTGTTTGGAAT<br>R: GGTGAGGTTGCAGAAACGATAAA
Human TLR6	F: TTCTCCGACGGAAATGAATTTGC<br>R: CAGCGGTAGGTCTTTTGGAAC
Human TLR7	F: TCCTTGGGGCTAGATGGTTTC<br>R: TCCACGATCACATGGTTCTTTG
Human TLR8	F: ATGTTCCTTCAGTCGTCAATGC<br>R: TTGCTGCACTCTGCAATAACT
Human TLR9	F: CTGCCTTCCTACCCTGTGAG<br>R: GGATGCGGTTGGAGGACAA
Human TLR10	F: AGGTTTGAGTGGGGCAAAAAT<br>R: CCATCACGCAAAAGAACCCAG
Human IDO	F: GCCAGCTTCGAGAAAGAGTTG<br>R: ATCCCAGAACTAGACGTGCAA
Human COX2	F: CTGGCGCTCAGCCATACAG<br>R: CGCACTTATACTGGTCAAATCCC
Human TSG6	F: TTTCTCTTGCTATGGGAAGACAC<br>R: GAGCTTGTATTTGCCAGACCG
Human IL6	F: ACTCACCTCTTCAGAACGAATTG<br>R: CCATCTTTGGAAGGTTCAGGTTG
Human IL8	F: TTTTGCCAAGGAGTGCTAAAGA<br>R: AACCCTCTGCACCCAGTTTTC
Human CCL2	F: CAGCCAGATGCAATCAATGCC<br>R: TGGAATCCTGAACCCACTTCT
Mouse TLR1	F: TTTGTCCCACAATGAGCTAAAGG<br>R: TTCTTTGCATATAGGCAGGGC
Mouse TLR2	F: CTCTTCAGCAAACGCTGTTCT<br>R: GGCGTCTCCCTCTATTGTATTG
Mouse TLR3	F: GTGAGATACAACGTAGCTGACTG<br>R: TCCTGCATCCAAGATAGCAAGT
Mouse TLR4	F: GCCTTTCAGGGAATTAAGCTCC<br>R: GATCAACCGATGGACGTGTAAA
Mouse TLR5	F: TGGGGACCCAGTATGCTAACT<br>R: CCACAGGAAAACAGCCGAAGT

Mouse TLR6	F: AGCCAAGACAGAAAACCCATC<br>R: GGGGTCATGCTTCCGACTAT
Mouse TLR7	F: ATGTGGACACGGAAGAGACAA<br>R: ACCATCGAAACCCAAAGACTC
Mouse TLR8	F: TTCCAGAAGCTATCCTTGTGACG<br>R: CATGCAGTTGACGATGGTTGC
Mouse TLR9	F: ATGGTTCTCCGTCGAAGGACT<br>R: GAGGCTTCAGCTCACAGGG
Mouse iNOS	F: GTTCTCAGCCCAACAATACAAGA<br>R: GTGGACGGGTCGATGTCAC
Mouse COX2	F: TTCCAATCCATGTCAAAACCGT<br>R: AGTCCGGGTACAGTCACACTT
Mouse IL6	F: CTGCAAGAGACTTCCATCCAG<br>R: AGTGGTATAGACAGGTCTGTTGG
Mouse IL12	F: CAATCACGCTACCTCCTCTTTT<br>R: CAGCAGTGCAGGAATAATGTTTC
